# Current knowledge in lentil genomics and its application for crop improvement

**DOI:** 10.3389/fpls.2015.00078

**Published:** 2015-02-23

**Authors:** Shiv Kumar, Karthika Rajendran, Jitendra Kumar, Aladdin Hamwieh, Michael Baum

**Affiliations:** ^1^Biodiversity and Integrated Gene Management Program, International Center for Agricultural Research in the Dry Areas, RabatMorocco; ^2^Division of Crop Improvement, Indian Institute of Pulses Research, KanpurIndia; ^3^International Center for Agricultural Research in the Dry Areas, CairoEgypt; ^4^International Center for Agricultural Research in the Dry Areas, AmmanJordan

**Keywords:** lentil, molecular markers, single nucleotide polymorphism (SNP), quantitative trait loci (QTL) mapping, marker assisted selection (MAS)

## Abstract

Most of the lentil growing countries face a certain set of abiotic and biotic stresses causing substantial reduction in crop growth, yield, and production. Until-to date, lentil breeders have used conventional plant breeding techniques of selection-recombination-selection cycle to develop improved cultivars.These techniques have been successful in mainstreaming some of the easy-to-manage monogenic traits. However, in case of complex quantitative traits, these conventional techniques are less precise. As most of the economic traits are complex, quantitative, and often influenced by environments and genotype–environment interaction, the genetic improvement of these traits becomes difficult. Genomics assisted breeding is relatively powerful and fast approach to develop high yielding varieties more suitable to adverse environmental conditions. New tools such as molecular markers and bioinformatics are expected to generate new knowledge and improve our understanding on the genetics of complex traits. In the past, the limited availability of genomic resources in lentil could not allow breeders to employ these tools in mainstream breeding program.The recent application of the next generation sequencing and genotyping by sequencing technologies has facilitated to speed up the lentil genome sequencing project and large discovery of genome-wide single nucleotide polymorphism (SNP) markers. Currently, several linkage maps have been developed in lentil through the use of expressed sequenced tag (EST) derived simple sequence repeat (SSR) and SNP markers.These maps have emerged as useful genomic resources to identify quantitative trait loci imparting tolerance to biotic and abiotic stresses in lentil. In this review, the current knowledge on available genomic resources and its application in lentil breeding program are discussed.

## INTRODUCTION

Lentil (*Lens culinaris* ssp. *culinaris* Medikus) is a diploid (2n=2X=14) self-pollinating crop with a genome size of approximately 4 Gbp (Arumuganathan and Earle, 1991). It provides affordable source of dietary proteins (22–35%), minerals, fiber, and carbohydrates to poor people and plays a vital role in alleviating malnutrition and micronutrient deficiencies in developing countries. As it exhibits low glycemic index, it is highly recommended by physicians for the people suffering from diabetes, obesity, and cardiovascular diseases (Srivastava and Vasishtha, 2012). In fact, vegetable protein is gaining preference over the animal protein for consumption by the health conscious people in the present day. This could be one of the reasons for increased per capita consumption (Vandenberg, 2009) and fivefold increase in global lentil production (from 0.85 to 4.43 Mt) during the last five decades, through a 155% increase in sown area and the doubling of average yields from 528 to 1068 kg ha^-1^ (FAOSTAT, 2014). Lentil cultivation often provides rotational benefits to cereal-based cropping systems through biological nitrogen fixation, carbon sequestration, and through effective control of weeds, diseases, and insect pests. It generates livelihood for the small-scale farmers practicing agriculture in the dryland agricultural ecosystems of South Asia, Sub-Saharan Africa, West Asia, and North Africa (Kumar et al., 2013). However, the lentil yields remain low in many developing countries as it is often cultivated as a rainfed crop under difficult edaphic conditions and subjected to terminal drought, heat stress, low soil fertility, and various diseases including ascochyta blight (*Ascochyta lentis*), fusarium wilt (*Fusarium oxysporum* f.sp. *lentis*), anthracnose (*Colletotrichum truncatum*), stemphylium blight (*Stemphylium botryosum*), rust (*Uromyces viciae-fabae*), collar rot (*Sclerotiun rolfsii*), root rot (*Rhizoctonia solani*), and white mold (*Sclerotinia sclerotiorum*) (Kumar et al., 2013; Sharpe et al., 2013). So far, the classical plant breeding approach of selection-recombination-selection has been successful in mainstreaming some of the easy-to-manage monogenic traits in lentil. However, this approach is less precise and time consuming when dealing with traits of breeders’ interest which are often quantitative in nature and highly influenced by environment and genotype–environment (GE) interaction (Kumar and Ali, 2006). In order to identify, fix, and select superior recombinants more precisely and efficiently, there is a need to integrate biotechnological approaches such as marker assisted selection (MAS) and genetic engineering in lentil breeding program to mainstream new genetic variability in the cultivated gene pool.

The current lentil breeding programs are limited in their ability to implement MAS due to a lack of genomic resources. In comparison to major legume crops such as soybean, common bean, pigeon pea, and chickpea, the pace of development of genomic resources is slow in lentil (Kumar et al., 2014). Large genome size, narrow genetic base, lack of candidate genes, low density linkage map, and the difficulty in identifying beneficial alleles are the main limiting factors in genomics enabled improvement in lentil. Molecular tools have occasionally been used by lentil breeders and geneticists to understand the genetic basis of a few traits related to biotic (ascochyta blight, anthracnose, rust, fusarium wilt, stemphylium blight) and abiotic (drought, frost, cold, boron, salinity) stresses (Kumar et al., 2014). Recent developments in the next generation sequencing (NGS) technologies have facilitated the development of array-based high-throughput (HTP) genotyping platforms with SNP markers. Bett et al. (2014) have carried out large amounts of next-generation sequencing on lentil cultivar, CDC Redberry. An initial draft of 23x coverage produced scaffolds covering over half the genome (2.7 Gb of the expected 4.3 Gb) and recent additional 125x coverage is currently being assembled. Gene sequences for several traits of interest were identified using the initial 23x draft assembly and derived SNP markers are now available for MAS in the lentil breeding program (Bett et al., 2014). Besides, the close phylogenetic relationships with the model legumes such as *Medicago truncatula* and *Lotus japonicus* have provided ample opportunities for comparative genome mapping and identified putative orthologous gene sequence resources in these genomes (Weller et al., 2012; Kaur et al., 2014). These genomic tools and technologies have opened up new avenues for practicing genomics assisted selection in lentil. There is also a tremendous scope to develop lentil cultivars through reverse genetic approaches. In this context, this review has been made to evaluate the research progress achieved in lentil genomics along with the discussion on future prospective for genetic enhancement.

## DEVELOPMENT OF GENOMICS RESOURCES

### MOLECULAR MARKERS

The first genetic map of lentil was constructed using morphological and isozyme markers in early 1980’s (Zamir and Ladizinsky, 1984; Tadmor et al., 1987). After the discovery of molecular markers starting from the restriction fragment length polymorphism (RFLP), significant progress has been made in molecular marker development and genotyping platforms in lentils. It began with the hybridization based DNA markers such as RFLP (Havey and Muehlbauer, 1989) and moved toward the use of PCR based markers such as random amplified polymorphic DNA (RAPD), amplified fragment length polymorphism (AFLP) and simple sequence repeats (SSR) markers for genotyping. The first comprehensive linkage map with 177 RAPD, AFLP, RFLP, and morphological markers was developed using interspecific recombinant inbred lines (RIL) population of a single cross of *L. culinaris* × *L. orientalis* (Eujayl et al., 1998a). Among the various PCR based markers, SSR markers have made significant contribution to the recent development of lentil genome maps. The first genomic library was constructed from a cultivated accession, ILL5588 using the restriction enzyme *Sau*3AI (*Staphylococcus aureus* 3A) and screened with (GT)10, (GA)10, (GC)10, (GAA)8, (TA)10, and (TAA) probes (Hamwieh et al., 2005). Using this library initially a set of 30 highly polymorphic SSR markers were developed. Since this study was aimed at isolating SSRs that are abundant and well distributed in the genome, a non-enriched library was used for screening purposes. Hamwieh et al. (2009) further developed an additional set of 14 SSR markers and used them for genetic diversity analysis of the lentil core set. A set of 122 functional SSR markers have recently been developed using a genomic library enriched for GA/CT motifs for utilization in the lentil breeding program (Verma et al., 2014).

Recently, the PCR-based markers are being rapidly replaced by the DNA chip based markers, particularly with SNPs. SNPs are abundant in nature and common even across legume genomes (Chagne et al., 2007). There are various technologies for evaluation of SNP loci and many of these are amenable to automation for allele calling and data collection. The availability of extensive sequence database has made a new beginning to exploit them as a HTP marker system for genome mapping studies. Recent efforts in re-sequencing alleles to discover SNPs in lentil have facilitated automated high-throughput genotyping platforms (HTP). As a result, SNPs have emerged as potential markers for NGS approaches. About 44,879 SNP markers have been identified in lentil using Illumina Genome Analyzer (Sharpe et al., 2013). Temel et al. (2014) have identified another set of 50,960 SNPs and constructed a SNP based linkage map in lentil. The recent discovery of high-density SNP markers has facilitated the establishment of ultra HTP genotyping technologies such as Illumina GoldenGate (GG), which can accommodate more than 1000 SNPs in GG platforms (Sharpe et al., 2013; Kaur et al., 2014). Since SNP discovery and genotyping require expensive and sophisticated platforms, the development and exploitation of SNP markers is still limited in lentil. There are techniques available to detect SNPs such as allele-specific PCR, single base extension and array hybridization methods. These are cost effective and through the use of allele-specific PCR (KASPar) markers, we can include small to moderate amount of SNPs for any specific application (Fedoruk et al., 2013; Sharpe et al., 2013).

### TRANSCRIPTOME ASSEMBLIES

As the characterization of lentil whole genome is still in progress, transcriptome assemblies provide excellent opportunities to identify expressed sequenced tag (EST) derived SSR and SNP markers and intron-targeted primers (ITP). In the early days, the classical dideoxynucleotide chain termination method of Sanger has been used to sequence cDNA libraries and generate ESTs across various crops. ESTs are short DNA sequences of 150–400 bp from a cDNA clone that correspond to a particular mRNA. Development of HTP functional genomics approaches like serial analysis of gene expression (SAGE) has led to the generation of more ESTs. The first EST library was made from a mixture of eight cultivars with varying seed phenotypes (Vijayan et al., 2009). The second cDNA library was prepared from the leaflets of a Canadian cultivar ‘Eston’ inoculated with *Colletotrichum truncatum* (Kumar et al., 2014). The cDNA clones corresponding to the ESTs of interest can be used as RFLP or CAPS based markers (Varshney et al., 2005). The EST sequence data also serve the purpose of identifying SSRs and/or SNPs. Before the ESTs, development of SSR and SNP markers was expensive and required high resource laboratories, but presently any user can download them from the database and use some special bioinformatic programs like MISA for SSR detection (Thiel et al., 2003; Varshney et al., 2005) and Snipper for SNP discovery (Kota et al., 2003; Varshney et al., 2005). As on January 2015, there are about 10,341 ESTs available for lentil (NCBI, 2015).

Kaur et al. (2011) carried out transcriptome sequencing of lentil based on the second-generation technology which permits large-scale unigene assembly and SSR marker discovery. They used tissue-specific cDNA samples from six genotypes (Northfield, ILL2024, Indianhead, Digger, ILL6788, and ILL7537) using Roche 454 GS-FLX Titanium technology, and generated c. 1.38 × 10^6^ ESTs. *De novo* assembly generated 15,354 contigs and 68,715 singletons. Out of huge ESTs produced, 3,470 SNP and EST-SSRs have been identified. Development of genomic resources has become cost effective with the advent of NGS of ESTs. Validation of a subset of 192 EST-SSR markers across a panel of 12 cultivated genotypes showed 47.5% polymorphism from a set of 2,393 EST-SSR markers developed in lentil (Kaur et al., 2011). In recent times, transcriptome cDNA library sequencing using Illumina GA/GAIIx system has provided a potential alternative. Sharpe et al. (2013) developed 3^′^-cDNA reads from nine *L. culinaris* and two *L. ervoides* accessions using 454 pyrosequencing technology, identified SNPs, selected the sub-set of SNP for the development of a 1536 SNP Illumina GG array and used the array to construct a SNP based genetic map of *L. culinaris* mapping population. Similarly, Verma et al. (2013) used the short reads obtained from Illumina GAII and developed *de novo* transcriptome assemblies of lentil, developed SSR markers and utilized them in diversity analysis. Temel et al. (2014) used two lentil cultivars, Precoz and WA8649041 and their RILs using Illumina CASAVA pipelines, detected SNP markers, and generated a SNP based linkage map. As a result of transcriptome sequencing, massive data have been obtained in the form of about 847,824 high quality sequence reads and the transcriptome assemblies with 84,074 unigenes (Sharpe et al., 2013; Verma et al., 2013).

### BI-PARENTAL MAPPING POPULATIONS

Efforts have been made at International Center for Agricultural Research in the Dry Areas (ICARDA) and national programs to develop mapping populations for key traits in lentil (**Table 1**). RIL populations have been developed from the crosses made between contrasting parents for the traits of interest through single seed descent method. Indian Institute of Pulses Research (IIPR) has recently developed RIL population from a cross between ILL6002 and ILL7663 in order to identify and map early growth vigor genes in lentil. Identification of markers linked to the gene(s)/QTL governing these traits will help in development of genotype having high biomass at early stage. For tagging and mapping of genes of earliness, another mapping population has been developed from a cross between Precoz (Medium early) and L4603 (early) at IIPR, Kanpur, India. Another mapping population segregating for earliness with a cross made between ILL5588 (late flowering) and ILL6005 (early flowering) is available in University of Tasmania, Hobart, TAS, Australia (Weller et al., 2012). It has the loci *ELF3* (EARLY FLOWERING 3) which involved in circadian clock function and contribute to reduce the photoperiod response in cultivars to be grown under short season environmental conditions. CSK Himachal Pradesh Agricultural University, Palampur, India has developed RIL populations involving both intra and intersubspecific crosses that differ for rust reaction, drought tolerance, flowering time, plant vigor, shattering tolerance, seed size, and seed weight. Two mapping populations one each with the University of Saskatchewan, Saskatoon, SK, Canada (ILL4605 × ILL5888) and PAU (L-9-12 × FLIP-2004-7L) have been used for molecular mapping (Saha et al., 2010b; Mekonnen et al., 2014). With the rapid generation advancement technology (Mobini et al., 2014) which allows 4–5 generations per year in lentil will boost the development of much needed genetic resources for genomics enabled improvement.

**Table 1:** Mapping populations developed for various traits in lentil at International Center for Agricultural Research in the Dry Areas (ICARDA).

Trait	Cross	Population size
Drought	ILL 7946 × ILL 7979	174
Cold	ILL4605 × ILL 10657	153
Earliness	ILL 7115 × ILL 8009	150
Rust	ILL 5888 × ILL 6002	152
Fusarium wilt	ILL213 × ILL5883, Precoz × Idleb 2	150
Zn content	ILL5722 × ILL9888	177
	ILL9888 × ILL5480	149
Fe content	ILL 9932 × ILL 9951	193

### GENETIC LINKAGE MAPS

In the past, both inter- and intra-specific mapping populations were used for the construction of linkage maps in lentil. The first genetic mapping (linkage analysis) was began by Zamir and Ladizinsky (1984) and the first map comprising DNA based markers was produced by Havey and Muehlbauer (1989). Subsequent maps were published by several workers. With the development of PCR based markers, the number of available markers across the *Lens* genome increased dramatically (Kumar et al., 2011, 2014). The first extensive map comprised of RAPD, AFLP, RFLP, and morphological markers was constructed using a RIL population from a cross between a cultivated *L. culinaris* ssp. *culinaris* cultivar and a *L. culinaris* ssp. *orientalis* accession (Eujayl et al., 1998a). As lentil has low level of polymorphism in the cultivated gene pool the inter-varietal linkage maps were developed through the use of diverge parents from the wild and cultivated species. However, such molecular maps derived from these populations often result low recombination rate and smaller map size. Intra-specific mapping populations have more practical utility in QTL identification and to tag desirable genes of interest than the previous kind of mapping population. Rubeena et al. (2003) published the first intraspecific lentil map comprising 114 RAPD, inter simple sequence repeat (ISSR) and resistance gene analog (RGA) markers. Rubeena et al. (2006) reported F_2_ map comprising 72 markers (38 RAPD, 30 AFLP, 3 ISSR, and one morphological) spanning 412.5 cM. The first *Lens* map to include SSR markers was that of Duran et al. (2004). Hamwieh et al. (2005) added 39 SSR and 50 AFLP markers to the map constructed by Eujayl et al. (1998a) to produce a comprehensive *Lens* map comprising 283 genetic markers covering 715 cM. Subsequently, the first lentil map that contained 18 SSR and 79 cross genera ITAP gene-based markers was constructed using a F_5_ RIL population developed from a cross between ILL5722 and ILL5588 (Phan et al., 2007). The map comprised seven linkage groups (LGs) that varied from 80.2 to 274.6 cM in length and spanned a total of 928.4 cM. Gupta et al. (2012a) used 196 markers including new 15 *M. truncatula* EST-SSR/SSR in a population of 94 RILs produced from a cross between ILL5588 and ILL5722 and generated 11 LGs covering 1156.4 cM. An intersubspecific F_2_ *Lens* linkage map consisting of 199 PCR-based markers (28 SSRs, 9 ISSRs and 162 RAPDs) mapped on to 11 LGs covering a distance of 3847 cM has been constructed (Gupta et al., 2012b). Recently, population specific linkage maps are developed by Perez de la Vega et al. (2011) and Andeden et al. (2013). A list of comprehensive linkage maps in lentil is provided in **Table 2**.

**Table 2:** List of molecular linkage maps developed in lentil.

Type of population	Parents	Population size	No. of loci	Type of markers	Map length (cM)	Reference
RIL	*Lens culinaris* ssp. *culinaris* × *L.c.*ssp*. orientalis*	14-80	20	Isozyme and four morphological markers	–	Tahir and Muehlbauer (1994)
F_2_	*L. culinaris* ssp. *culinaris* × *L.c.*ssp*. orientalis*		10	Isozymes	–	Zamir and Ladizinsky (1984)
F_3_	*L. culinaris* × *L. ervoides* and *L. culinaris* × *L. ervoides*	10722–56	18	Isozymes	258	Tadmor et al. (1987)
RIL	*L.c.*ssp*. orientalis* × *L. culinaris* ssp*. culinaris*	86	177	RAPD, AFLP, RFLP, and morphological markers	1073	Eujayl et al. (1998a)
F_2_	*L. culinaris* ssp. *culinaris* × *L.c.*ssp*. orientalis*	113	200	RAPD, ISSR, AFLP, SSR, CAPS, SRAPS, and morphological markers	2234	Duran et al. (2004), Fratini et al. (2004), de la Puente et al. (2013)
RIL	ILL5588 × L692-16-1 (s)	86	283	SSR, AFLP	751	Hamwieh et al. (2005)
F_2_	ILL5588 × ILL7537	150	114	RAPD, ISSR, and RGA	784	Rubeena et al. (2003)
RIL	Eston × PI 320937	94	207	AFLP, RAPD, and SSR	1868	Tullu et al. (2006, 2008)
RIL	Precoz × WA 8649041	94	166	AFLP, ISSR, RAPD, and morphological markers	1396	Tanyolac et al. (2010)
RIL	ILL 6002 × ILL 5888	206	139	SSR, RAPD, SRAP, and morphological markers	1565	Saha et al. (2010a, 2013)
RIL	WA8649090 × Precoz	106	130	RAPD, ISSR, and AFLP	1192	Kahraman et al. (2004, 2010)
RIL	ILL5722 × ILL5588	94	211	RAPD, ISSR, ITAP, and SSR	1392	Gupta et al. (2012a)
F_2_	L830 × ILWL77	114	199	SSR, ISSR, and RAPD	3843	Gupta et al. (2012b)
RIL	CDC Robin × 964a-46	139	561	SNP, SSR, and seed color genes	697	Fedoruk et al. (2013), Sharpe et al. (2013)
RIL	Cassab × ILL 2024	126	318	SSR and SNP	1178	Kaur et al. (2014)
RIL	PI 320937 × Eston	96	194	AFLP, SSR, and SNP	840	Sever et al. (2014)
RIL	Precoz × WA 8649041	101	519	SNP	540	Temel et al. (2014)
RIL	ILL 8006 × CDC Milestone	–	149	AFLP, SSR, and SNP	497	Aldemir et al. (2014)

### COMPARATIVE GENOME MAPPING

Comparative genome mapping has demonstrated different levels of genome conservation among crop species during the course of evolution (Choi et al., 2004; Zhu et al., 2005). The lentil genome has shown different degrees of synteny with other legume crops (Weeden et al., 1992; Simon and Muehlbauer, 1997; Phan et al., 2007; Choudhary et al., 2009). Development of PCR-based markers has improved transferability of genetic information among species through comparative genomics and has facilitated the establishment of phylogenetic relationship in plants species. Since the availability of SSR markers in lentil is limited, other legumes offer great scope of marker transferability for genome-wide coverage. Pandian et al. (2000) observed 5% transferability of chickpea-specific STMS primers in lentil while Reddy et al. (2010) observed successful amplification of 62% *Trifolium* markers followed by *Medicago* (36%) and *Pisum* (25%). Datta et al. (2011) reported transferability of 19 STMS markers in lentil from common bean, chickpea, pigeon pea, and soybean. The lack of lentil-specific SSR markers propelled the mining and transfer of EST-SSR sequences from the model genome *M. truncatula* to enrich an existing intraspecific lentil genetic map (Gupta et al., 2012a). They published 21 clear and reproducible SSR markers showing polymorphism between parents, Northfield and Digger. EST-based ITAP markers have recently been developed from related crops and applied to lentil. ESTs were compared for phylogenetic distant from *M. truncatula*, *Lupinus albus,* and *G. max* to produce 500 ITAP markers that could be applied to lentil (Phan et al., 2007). Also, 126 *M. truncatula* cross-species markers were used to generate comparative genetic maps of lentil and white lupin and macrosyntenic relationships between lentil and field pea was observed. The techniques of comparative genomics provided significant opportunities for genetic diversity studies in lentil. The conserved primers (CPs) based on *M. truncatula* EST sequences flanking one or more introns were used to sequence amplicons in 175 wild and 133 domesticated lentil accessions (Alo et al., 2011). The analysis of the sequences confirmed that *L. nigricans* and *L. ervoides* are well-defined between the species at the DNA sequence level. The availability of draft genome sequences of *M. truncatula*, *L. japonicus,* and *Glycine max* have increased the possibilities of deriving more genomic resources by exploring new molecular markers through bioinformatics platforms which are capable of transfer across the species, belong to the Galegoid clade. Weller et al. (2012) identified two major loci controlling differences in photoperiod response between wild and domesticated pea *HR* (High response to photoperiod) and *ELF3* and identified orthologous gene loci of *ELF3* in lentil. Recently, Kaur et al. (2014) made a comparison of the flanking markers SNP_20002998 and SNP_20000246 in lentil for boron tolerance with the *Arabidopsis thaliana* and *M. trucatuala* genome sequences and identified candidate genes associated with boron tolerance.

### FUNCTIONAL GENOMICS

Genomic maps are useful to identify gene(s)/QTL responsible for controlling the variation for the underlying trait of interest. Gene cloning approach helps to characterize and reveal the function of the gene/QTL being identified. The knowledge of genes cloned in legumes can facilitate the development of functional markers for MAS. Many functionally known resistance gene analogs (RGA) have been cloned in lentil (Yaish et al., 2004). Likewise the numerous genes coding transcription factors (TFs) are identified in *Arabidopsis* in a large scale. As the distribution of TF genes does not significantly differ between legume and non-legume species, TF genes have been identified in legumes on the basis of sequence homology with *Arabidopsis* genes. Using functional genomics approaches, genes expressing differentially in contrasting genotypes can also be identified. Differential gene transcript profiles were assessed among resistant (ILL7537) and susceptible (ILL6002) lentil genotypes at 6, 24, 48, 72, and 96 h after inoculation with *Ascochyta lentis* (AL4 isolate; Ford et al., 2007). The non-redundant differentially expressed genes for each accession and time points were hierarchically clustered using Euclidean metrics. In total, 25 differentially expressed sequences were up-regulated and 56 down-regulated in ILL7537 whereas 26 were up-regulated and 44 down-regulated in ILL6002. Several candidate defense genes were characterized from lentil including a *b*-1, 3-glucanase, a pathogenesis-related protein from the Bet v I family, a pea disease resistance response protein 230 (DRR230-a), a disease resistance response protein (DRRG49-C), a PR4 type gene and a gene encoding an antimicrobial SNAKIN2 protein, all of which have been fully sequenced. Several TFs were also recovered at 6 h after inoculation and future aim is to further biologically characterize these and earlier responses to gain a comprehensive understanding of the key pathogen recognition and defense pathways to *A. lentis* in lentil. Also, the full-length gene sequences will be used in transgenic studies to further characterize their functions. Microarrays play important role in identifying gene networks underlying the expression of important plant traits. A DNA pulse chip made up of 565 ESTs from a chickpea cDNA library enriched for reaction to *A. rabiei*, 156 ESTs from a *Lathyrus* cDNA library enriched for reaction to *A. pinodes* and 41 lentil ESTs and RGAs from the GenBank database (Coram and Pang, 2005) was employed to study expression profiles for ascochyta blight resistant (ILL7537) and susceptible (ILL6002) cultivars (Mustafa et al., 2006).

## APPLICATION OF GENOMIC RESOURCES FOR LENTIL IMPROVEMENT

### GENETIC FINGER PRINTING

**FIGURE 1:**
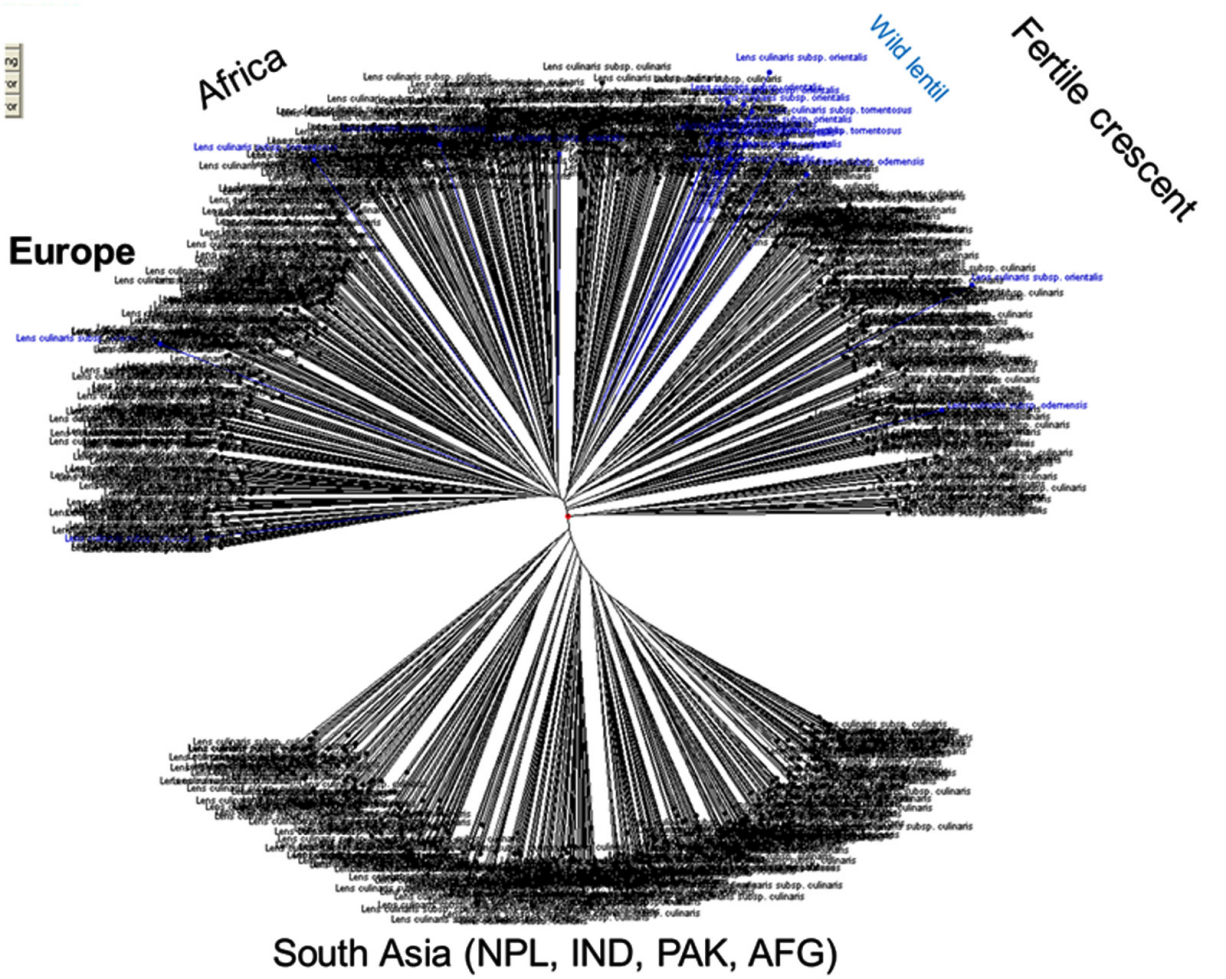
**Cluster analysis of core ICARDA lentil (both wild and cultivated) germplasm collections using 22 SSR markers.** The results indicated two major clusters separating south Asia (Nepal, India, Pakistan and Afghanistan) from the Middle East and western countries (adapted from Kumar et al., 2014).

**FIGURE 2:**
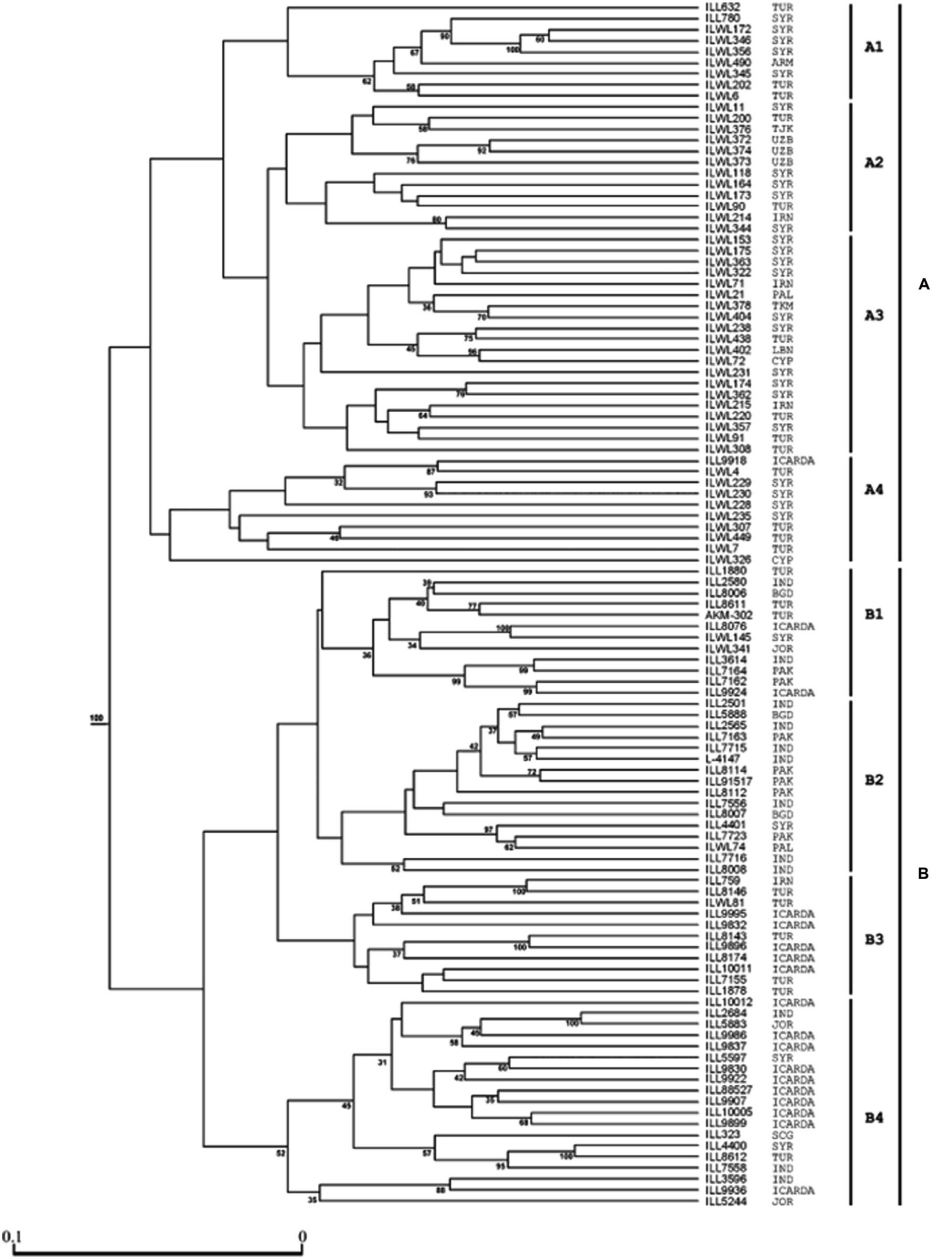
**Cluster analysis of ICARDA lentil (both wild and cultivated) mini-core set lentil accessions using 14 SSR markers.** The groups are denoted on the right side as A or B, and the sub-groups as A1, A2, A3, A4, B1, B2, B3, and B4. The origins of 109 lentil accessions are listed closed to the genotype numbers. Bootstrap values of above 30% are indicated at the nodes. The abbreviations of the countries: Bangladesh (BGD), India (IND), Iran (IRN), Jordan (JOR), Pakistan (PAK), Syria (SYR), Turkey (TUR), Serbia & Montenegro (SCG), Palestine (PAL), Armenia (ARM), Cyprus (CYP), Uzbekistan (UZB), Tajikistan (TJK), Turkmenistan (TKM), Lebanon (LBN) (adapted from Hamwieh et al., 2009; Kumar et al., 2014).

Genetic diversity analysis has been studied among a set of cultivated and wild lentils using various molecular marker system and genetic materials. Earlier studies have used RFLP, AFLP, and RAPD markers to assess genetic diversity and phylogenetic analyses within and among *Lens* species (Havey and Muehlbauer, 1989; Aboelwafa et al., 1995; Sharma et al., 1995, 1996; Ahmad and McNeil, 1996; Ford et al., 1997) and gene mapping (Eujayl et al., 1998b; Tullu et al., 2003; Duran et al., 2004; Kahraman et al., 2004; Hamwieh et al., 2005). As a part of the CGIAR’s Generation Challenge Program (GCP), ICARDA has identified a composite collection of lentil germplasm and characterized them by using SSR markers. ICARDA holds the largest global collection of lentil with >11,000 accessions. From this collection, a global composite collection of 960 accessions (**Table 3**) representing landraces, wild relatives, elite breeding lines, and cultivars was established (Furman, 2006). The results indicated two major clusters separating south Asia (Nepal, India, Pakistan, and Afghanistan) from the Middle East and western countries (**Figure 1**). The major output of this study was a reference set which represents around 15% (135 accessions) of the global composite collection representing all the geographical regions. This set has been phenotyped for different biotic and abiotic stresses, and emerged as a useful genetic resource to start with (Kumar et al., 2014). Recently, a set of SSR markers was used to study the genetic diversity of lentil mini core set. The mini core collection comprised 109 accessions from 15 countries representing 57 cultigens (including 18 breeding lines) from 8 countries to 52 wild accessions (*L*. *culinaris* ssp. *orientalis*, *L*. *culinaris* ssp. *tomentosus* and *L. culinaris* ssp. *odemensis*) from 11 countries. The total alleles detected across the SSR loci were 182, with a mean of 13 alleles per locus. Wild accessions were rich in allelic variation (151 alleles) compared to cultigens (114 alleles). The genetic diversity index for the SSR loci in the wild accessions ranged from 0.16 (SSR28 in *L. culinaris* ssp. *odemensis*) to 0.93 (SSR66 in *L. culinaris* ssp. *orientalis*) with a mean of 0.66, while in the cultigens, genetic diversity varied between 0.03 (SSR28) and 0.87 (SSR207) with a mean of 0.65. Cluster analysis indicated two major clusters (**Figure 2**), mainly one with the cultigens and the other with wild accessions (Hamwieh et al., 2009). The recent techniques of comparative genomics also provided significant opportunities for genetic diversity studies in lentil. The CPs based on *M. truncatula* EST sequences flanking one or more introns were used to sequence amplicons in 175 wild and 133 domesticated accessions. This analysis of the sequences confirmed that *L. nigricans* and *L. ervoides* are well-defined species at the DNA sequence level. *L. culinaris* ssp. *orientalis* is the progenitor of domesticated lentil, *L. culinaris* ssp. *culinaris*, but a more specific area of origin can be suggested in southern Turkey. The study detected the divergence, following domestication, of the domesticated gene pool into overlapping large seeded (megasperma) and small-seeded (microsperma) groups and observed that lentil domestication led to a loss of genetic diversity of approximately 40% (Alo et al., 2011).

**Table 3:** Composition of core germplasm representing 10% of the global lentil collection by ICARDA.

Country	No. of accessions	Country	No. of accessions	Country	No. of accessions
Afghanistan	30	Germany	10	Romania	2
Albania	1	Greece	17	Russian	13
Algeria	11	Guatemala	1	Saudi Arabia	1
Argentina	6	Hungary	3	Scg	4
Armenia	3	India	192	Slovakia	1
Azerbaijan	4	Iran	103	Spain	17
Bangladesh	6	Iraq	11	Sudan	2
Belgium	1	Italy	6	Syria	70
Brazil	2	Jordan	46	Tajikistan	5
Breeding	35	Lebanon	9	Tunisia	8
Bulgaria	6	Libyan	1	Turkey	69
Canada	3	Macedonia	3	Turkmenistan	1
Chile	27	Mexico	8	Ukraine	5
China	1	Morocco	14	US	10
Colombia	3	Nepal	28	Unknown	7
Croatia	1	Netherlands	1	Uruguay	1
Cyprus	9	Norway	1	Uzbekistan	2
Czech Republic	6	Pakistan	27	Yemen	12
Egypt	25	Pal	4	Yugoslavia	2
Ethiopia	49	Poland	4	**Sum**	**960**
France	5	Portugal	5		

### HYBRID TESTING

Making crosses between diverse parents is difficult in practice in lentil because of very small flowers leading to increase the chances of selfing. In addition to this, differentiating F_1_ plants from selfed ones also becomes difficult due to low phenotypic diversity between the parents. Hence molecular markers have been found very useful to detect the hybridity of F_1_ plants in lentil. Solanki et al. (2010) used molecular markers in lentil and detected only 21% plants as true hybrids. These results suggest that molecular markers can reduce the time and money required to grow a population from selfed or admixed plants and increase the efficiency of plant breeders in selection of recombinant plants.

### MARKER ASSISTED SELECTION

Molecular markers linked to desirable gene(s)/QTL have been reported for marker-assisted selection in lentil (**Table 4**). Morphological markers viz., cotyledon (*Yc*), anthocyanin in stem (*Gs*), pod indehiscence (*Pi*), seed coat pattern (*Scp*), flower color (*W*), radiation frost tolerance locus (*Rf*), early flowering (*Sn*), and ground color of the seed (*Gc*) were mapped as qualitative markers because they exhibited monogenic dominant mode of inheritance (Eujayl et al., 1998a; Duran et al., 2004; Hamwieh et al., 2005; Tullu et al., 2008). Further analysis for the association between DNA markers and Fusarium wilt resistance (Fw) gene was confirmed (Eujayl et al., 1998b; Hamwieh et al., 2005). However, only SSR59-2B was closely linked with Fw at 19.7 cM (Hamwieh et al., 2005). Anthracnose disease resistance (Lct-2) was mapped by Tullu et al. (2003). To date, quantitatively inherited traits have been mapped by Duran et al. (2004) who detected five QTL each for the height of the first ramification and flowering time, three for plant height, seven for pod dehiscence, and one each for shoot number and seed diameter. Five and four QTL were identified for winter survival and winter injury, using a RIL population of 106 lines derived from WA8649090 × Precoz (Kahraman et al., 2004). In this study, experiments were conducted at multiple locations and only one of five QTL was expressed in all environments. Mapping of *Ascochyta* blight resistance using an F_2_ population derived from ILL7537 × ILL6002 identified three QTL accounting for 47% (*QTL*-1 and *QTL*-2) and 10% (*QTL*-3) of disease variation. Recently, QTL conferring resistance to *Stemphylium* blight and rust diseases using RIL populations were identified in lentil (Saha et al., 2010a,b). Though the use of F_2_ populations in identification of QTL has been done widely in lentil, their use in marker-trait analysis has led to identification of only major QTL. Thus, several minor QTL were overlooked in such populations and identification of environmental responsive QTL was difficult. Because quantitative traits are influenced by both genetic and environmental effects, RILs or near isogenic lines (NILs) are more suitable populations to accurately dissect their components. For ascochyta blight, three QTL each were detected for resistance at seedling and pod/maturity stages (Gupta et al., 2012a). Together these accounted for 34 and 61% of the total estimated phenotypic variation and demonstrated that resistance at different growth stages is potentially conditioned by different genomic regions. Kaur et al. (2014) identified QTL for boron tolerance in Cassab × ILL2024 mapping population. Both simple interval mapping (SIM) and composite interval mapping (CIM) confirmed the presence of QTL in LG4.2 between SNP_20002998 and SNP_20000246. The flanking markers identified may be useful for MAS and pyramiding of potentially different resistance genes into elite backgrounds that are resistant throughout the cropping season. While using QTL pyramiding approach Taran et al. (2003) identified lines with combined resistance to ascochyta blight resistance (*AbR1* and *ral1*) and Anthracnose (OPO6_1250_) in CDC Robin and 964a-46 RIL population for developing cultivars resistance to both ascochyta blight and anthracnose in lentil.

**Table 4:** Molecular markers linked to desirable genes/QTL for marker-assisted selection in lentil.

Traits	Mapping population	Marker linked with the QTL	Phenotypic variation explained by the QTL (%)	Reference
*Ascochyta* blight resistance	ILL5588 × ILL6000	RAPD	90	Ford et al. (1999)
	ILL5588 × ILL7537 and ILL7537 × ILL6002	RAPD, AFLP, and ISSR	Up to 50	Rubeena et al. (2006)
	Eston × PI320937	AFLP and RAPD	41	Tullu et al. (2006)
	NorthWeld (ILL5588) × Digger (ILL5722)	ITAP, SSR, and ISSR	Up to 61	Gupta et al. (2012a)
Earliness	Eston × PI320937	RAPD, AFLP, and SSR	37–46	Tullu et al. (2008)
Plant height	Eston × PI320937	RAPD, AFLP, and SSR	31–40	Tullu et al. (2008)
	*L. culinaris* ssp. *culinaris* × *L.c.*ssp. *orientalis*	RAPD, ISSR, AFLP, SSR, and morphological markers	38.2	Fratini et al. (2007)
Branches at the first node	*L. culinaris* ssp. *culinaris* × *L.c.*ssp. *orientalis*	RAPD, ISSR, AFLP, SSR, and morphological markers	91.7	Fratini et al. (2007)
Total number of branches	*L. culinaris* ssp. *culinaris* × *L.c.*ssp. *orientalis*	RAPD, ISSR, AFLP, SSR and morphological markers	54	Fratini et al. (2007)
Height at the first node	*L. culinaris* ssp. *culinaris* × *L.c.*ssp. *orientalis*	RAPD, ISSR, AFLP, SSR, and morphological markers	33.3	Fratini et al. (2007)
Flowering time	*L. culinaris* ssp. *culinaris* × *L.c.*ssp. *orientalis*	RAPD, ISSR, AFLP, SSR, and morphological markers	90.4	Fratini et al. (2007)
Pod dehiscence	*L. culinaris* ssp. *culinaris* × *L.c.*ssp. *orientalis*	RAPD, ISSR, AFLP, SSR, and morphological markers	81.3	Fratini et al. (2007)
Seed weight	*L. culinaris* ssp. *culinaris* × *L.c.*ssp. *orientalis*	RAPD, ISSR, AFLP, SSR, and morphological markers	18.2	Fratini et al. (2007)
Seed diameter	*L. culinaris* ssp. *culinaris* × *L.c.*ssp. *orientalis*	RAPD, ISSR, AFLP, SSR, and morphological markers	37	Fratini et al. (2007)
Winter hardiness	WA8649090 × Precoz	RAPD, ISSR, and AFLP	20.45	Kahraman et al. (2010)
Cotyledon color class (Yc)	CDC Robin × 964a-46	SNP, SSR, and seed color loci	23	Fedoruk et al. (2013)
Seed thickness	CDC Robin × 964a-46	SNP, SSR, and seed color loci	8.4	Fedoruk et al. (2013)
Seed diameter	CDC Robin × 964a-46	SNP, SSR, and seed color loci	Up to 60	Fedoruk et al. (2013)
Seed plumpness	CDC Robin × 964a-46	SNP, SSR, and seed color loci	Up to 50	Fedoruk et al. (2013)
Days to 50% flowering	CDC Robin × 964a-46	SNP, SSR, and seed color loci	Up to 34	Fedoruk et al. (2013)
	ILL6002 × ILL5888	SSR, SRAP, RAPD	24.2	Saha et al. (2013)
Hundred seed weight	ILL6002 × ILL5888	SSR, SRAP, RAPD	17.5	Saha et al. (2013)
Plant height	ILL6002 × ILL5888	SSR, SRAP, RAPD	15.3	Saha et al. (2013)
Seed diameter	ILL6002 × ILL5888	SSR, SRAP, RAPD	32.6	Saha et al. (2013)
*Stemphylium* blight resistance	ILL6002 × ILL5888	SSR, SRAP, RAPD	46	Saha et al. (2010a)
Boron tolerance	Cassab × ILL2024	SNP	71	Kaur et al. (2014)

### GENE-TRAIT ASSOCIATION ANALYSIS USING NATURAL DIVERSE POPULATION

Bi-parental mapping approach causes more chances for segregation distortion through favoring of one parental allele over another. Also, the molecular markers which can be polymorphic within the interspecific populations might not be polymorphic at the species level as genetic background affects their utility in MAS process. Association mapping is an alternative approach that can address these shortcomings of bi-parental linkage mapping. While using historical recombination in natural populations, landraces, breeding material and varieties, association mapping does marker-trait association and identifies QTL with high resolution. There are two different types of association mapping which can be done on any crop species: genome-wide association studies (GWAS) and candidate gene association mapping. However, to date there are very few reported studies about association mapping in lentil. It is mainly due to the lack of genomic resources available for lentil. After identification of 1536-SNP Illumina GG array (Lc1536) by Sharpe et al. (2013), the Lc1536 array was used in GWAS. The linkage disequilibrium (LD) in lentil may occur similar to that in barley, soybean, and *M. truncatula* (Branca et al., 2011). Fedoruk et al. (2013) used association mapping in lentil to identify QTL for seed size and seed shape. As the properly designed association panels have a greater frequency of alleles encompassing the genetic variation of a crop, it can greatly facilitate to save time and cost while performing MAS in lentil.

### GENETIC TRANSFORMATIONS

Transgenic approach uses functional genes which are not available within the crossable gene pool. Thus cloned genes are important genomic resources for making genetic manipulation through transformation. Commonly, the particle bombardment and the *Agrobacterium tumefaciens* infection methods have been used to introduce genes with novel functions. With the explosion of sequence information available in the databases, transformation systems have also become useful tools to study gene function via RNA interference ‘knockout,’ T-DNA insertion or transforming a genotype lacking a particular gene. Thus a robust, reproducible and efficient transformation system combined with a protocol to regenerate complete fertile plants from transformed cells is essential to fully study plant gene functions.

Following the initial report of shoot regeneration (Bajaj and Dhanju, 1979) from apical meristems, it has been achieved routinely with different explants such as apical meristems (Bajaj and Dhanju, 1979), stem nodes (Polanco et al., 1988; Singh and Raghuvanshi, 1989; Ahmad et al., 1997), cotyledonary node (Warkentin and McHughen, 1992), epicotyls (Williams and McHughen, 1986), decapitated embryo, embryo axis and immature seeds (Polanco and Ruiz, 2001), and cotyledonary petioles (Khawar and Özcan, 2002). The induction of functional roots on *in vitro*-developed shoots has been the major challenge in lentil micro propagation. The difficulty to induce roots is thought to be associated with the use of cytokinin to obtain multiple shoots from the initial explants (Mohamed et al., 1992). Among the several studies conducted on root induction from shoots, Fratini and Ruiz (2003) reported 95% rooting efficiency from nodal segments cultured in an inverted orientation in media with 5 μM indole acetic acid (IAA) and 1 μM kinetin (KN). Sarker et al. (2003) reported 30% rooting efficiency on MS medium supplemented with 25 mg/l indole butyric acid (IBA).

To date, transformation of lentil has been reported through *A. tumefaciens*-mediated gene transfer (Lurquin et al., 1998) and biolistic transformation including electroporation (Chowrira et al., 1996) and particle bombardment (Gulati et al., 2002; Mahmoudian et al., 2002). Warkentin and McHughen (1992) reported the susceptibility of lentil to *A. tumefaciens* and later evaluated a number of explant types including shoot apices, epicotyl, root, cotyledons, and cotyledonary nodes. All explants showed transient *b*-glucuronidase (GUS) expression at the wound sites except cotyledonary nodes, which were subsequently transformed by Sarker et al. (2003). Oktem et al. (1999) reported the first transient and stable chimeric transgene expression on cotyledonary lentil nodes using particle bombardment. Gulati et al. (2002) reported regeneration of the first fertile transgenic lentil plants on MS medium with 4.4 μM benzyladenine (BA), 5.2 μM gibberellic acid (GA3), and chlorsulfuron (5 nM for 28 days and 2.5 nM for the rest of the culture period), followed by micrografting and transplantation in soil. The first successful work was reported by Barton et al. (1997), using pCGP1258 plasmid construct on four lentil genotypes. Khatib et al. (2007) have developed herbicide-resistant lentil through *A. tumefaciens* mediated transformation. This was achieved with the same plasmid construct pCGP1258, harboring the *bar* gene conferring resistance to the herbicide glufosinate ammonium that was transformed using *A. tumefaciens* strain *AgL*0. Three lentil lines, ILL5582, ILL5883, and ILL5588, were used and a high selection pressure of 20 mg/l of glufosinate was applied to the explants for 18 weeks. Surviving shoots were subsequently grafted onto non-transgenic rootstock and plantlets were transferred to soil and acclimatized. The presence of the transgene was confirmed by PCR and the gene function was confirmed via herbicide application. Recently, Akcay et al. (2009) reported the production of transgenic lentil plants via *Agrobacterium*-mediated transformation and the stable transmission of the *nptII* and *gus*A genes in the subsequent generations. However, these studies were mostly confined to establish transformation techniques rather than the introduction of genes into improved varieties. Khatib et al. (2011) reported for the first time the introduction of the *DREB1A* gene into lentil for enhancing drought and salinity tolerance. The PCR results confirmed the insertion and stable inheritance of the gene of interest and *bar* marker gene in the plant genome. The Southern blot analysis revealed integration of a single copy of the transgene. The *DREB1A* gene driven by rd29A promoter transcribed in the transgenic plants by inducing salt stress in form of sodium chloride solution. The results showed that mRNA was accumulated and thus the *DREB1A* gene was expressed in the transgenic plants.

## FUTURE PERSPECTIVES

Application of MAS is still limited in lentil. The NGS technology has opened up new opportunity for the fast development of sequence based markers. Access to HTP genotyping and sequencing technologies is expected to speed up the genetic gain across the target environments in lentil. These developments ultimately will increase the utilization of genomic resources in genetic improvement of lentil and will lead fast track development of improved cultivars. Further, increasing number of re-sequencing database in coming days will allow identification of more SNPs and consequently, HTP cost-effective genotyping assays using only informative SNPs would become available for the development of high density linkages for MAS. Recent collaborations among the labs in Canada, Australia, Czech Republic, Spain, USA, ICARDA, and Kenya will facilitate further assembly and annotation of the draft genome, as well as add to the growing database of genetic diversity in the global lentil germplasm. This will include use of long reads based on PacBio sequencing to assemble smaller scaffolds into larger assemblies. Key mapping populations would be genotyped using GBS technology to anchor scaffolds into chromosomal pseudo-molecules and selected lentil genotypes need to be re-sequenced to reveal the genomic diversity in lentil germplasm and provide a road map for future breeding activities. These advances also simultaneously encourage the lentil breeders to develop specialized mapping population such as nested association mapping (NAM) and multi-parents advanced generation inter-cross (MAGIC) populations to generate the genome-wide allelic and haplotype data. Likewise, non-transgenic techniques such as target-induced local lesion in genomes (TILLING) and RNA interference (RNAi) also have demonstrated potential scope for lentil improvement. TILLING has significantly contributed to the understanding of function of pea subtilase (*SBT1.1*) and tendril-less (*tl*) genes which control the seed size and tendril formation (D’Erfurth et al., 2012). At ICARDA, mutagenic lentil populations have been recently developed using the mutagen, ethyl methane sulfonate (EMS) in order to identify any point and knock-out mutations for tendril formation and other traits such as pod shattering, herbicide tolerance and *Orobanche* tolerance. Likewise the other non-transgenic approaches including RNAi technology and virus-induced gene silencing (VIGS) will help understand the molecular mechanisms of biological nitrogen fixation in lentil. The coming years would provide more opportunities to integrate GAB tools in the conventional breeding program. At the same time, more concerted efforts are required to develop other genomic resources such as BAC libraries and other transcriptome assemblies.

## CONCLUSION

Identifying the desired variability for target traits, utilizing the variability in breeding programs, and selecting and advancing the targeted recombinants are the major steps in a breeding program. Conventional breeding approaches are helpful to utilize the available genetic variability in the cultivated germplasm, resulting in the development of several red and yellow cotyledon varieties of lentil with tolerance/resistance to cold, ascochyta blight, rust, and wilt. In the last decade, several linkage maps have been developed and QTL/genes identified for the traits of interest in lentil. This has opened up the scope for mainstreaming genomics enabled improvement in lentil breeding programs. It will get further boost once the draft genome sequence and resequencing of the reference set of lentil is completed.

## Conflict of Interest Statement

The authors declare that the research was conducted in the absence of any commercial or financial relationships that could be construed as a potential conflict of interest.
